# Therapeutic plasma exchange in patients with COVID-19 pneumonia in intensive care unit: a retrospective study

**DOI:** 10.1186/s13054-020-03215-8

**Published:** 2020-08-08

**Authors:** Bulent Gucyetmez, Hakan Korkut Atalan, Ibrahim Sertdemir, Ulkem Cakir, Lutfi Telci, COVID-19 Study Group, Aylin Ogan, Aylin Cimet Ayyildiz, Berrin Yalcin, Behiye Oren, Fadıl Havas, Sevda Dizi, Birsen Kose, Umran Yakici, Cenk Sahan, Elif Ozkilitci, Ugur Tunali, Deniz Gunes, Ozlem Dincer, Reyhan Sahin, Duran Ozdemir, Mehtap Selcuk, Ceyhun Solakoglu, Unsal Arif Turan, Erkan Kaya, Mustafa Emre Kavlak, Pelin Katar, Hande Aygun, Kerim Cikim, Ozkan Uysal, Nur Ozturk Kaskir, Aysun Soylu

**Affiliations:** 1 Department of Anesthesiology and Reanimation, Acibadem Mehmet Ali Aydinlar University School of Medicine, Kerem Aydınlar Kampüsü, Kayışdağı Cad No:32 Ataşehir, 34752 Istanbul, Turkey; 2 General Intensive Care Unit, Acibadem International Hospital, Istanbul, Turkey; 3 General Intensive Care Unit, Memorial Atasehir Hospital, Istanbul, Turkey; 4 Department of Biostatistics and Bioinformatics, Institute of Health Sciences, Acibadem Mehmet Ali Aydinlar University, Istanbul, Turkey; 5 Department of Nephrology, Acibadem Mehmet Ali Aydinlar University School of Medicine, Istanbul, Turkey; 6 General Intensive Care Unit, Acibadem Atakent Hospital, Istanbul, Turkey; 7 General Intensive Care Unit, Acibadem Bakirkoy Hospital, Istanbul, Turkey

In patients with COVID-19 pneumonia, high risk of thrombosis became a current issue, and d-dimer levels indicating fibrin degradation products (FDPs) in the plasma were found as a predictor for mortality [1, 2]. Although unfractionated heparin (UFH) and low-molecular-weight heparin (LMWH) decrease the production of FDPs by inhibiting factors Xa and II, they cannot contribute metabolization of existing FDPs. Furthermore, FDPs cannot be filtered by known cytokine filters because of their molecular weight (minimum 240 kDa) [3, 4]. Yet, FDPs can be removed by therapeutic plasma exchange (TPE) [5]. Therefore, recently, three consecutive TPE sessions were performed in selected patients with COVID-19 pneumonia in intensive care units (ICUs) after the assessment of their clinical and coagulation status. In the study, the effect of TPE on outcomes was retrospectively investigated in patients with COVID-19 pneumonia.

All COVID-19 patients admitted to 5 different tertiary ICUs between 10 March and 10 May 2020 were evaluated, and 73 of 91 patients were included in the study. The patients who died within the first 4 days and who were still in the ICUs on 10 May were excluded. According to the Turkish Health Minister Algorithm for COVID-19, all included patients received the same antiviral (favipiravir, hydroxychloroquine, azithromycin) therapy and anticoagulant prophylaxis (UFH infusion 100 mcg/kg or LMWH 0.01 mL/kg). Since two different protocols were used in 5 ICUs, patients with d-dimer ≥ 2 in 3 ICUs had only received therapeutic anticoagulation whereas patients with d-dimer ≥ 2 in the other 2 ICUs had received TPE plus therapeutic anticoagulation. In all ICUs, for all patients in GII, echocardiography, lower extremity venous Doppler, and, if pulmonary thrombosis suspected, thorax computerized tomography angiography were performed. After collecting data, 73 patients were divided into 2 groups as group I (GI) (d-dimer < 2 mg/L) and group II (GII) (d-dimer ≥ 2 mg/L), and then GII was also divided into 2 groups as GIIa (TPE+) and GIIb (TPE−). Patients’ characteristics, respiratory and laboratory parameters, and outcomes were recorded. Propensity score matching (PSM) analysis was conducted on R v4.0.1 (0.2 caliper without replacement and nearest neighbor model, 1:1 ratio) by using 14 covariates (age, gender, CCI, APACHE II, SOFA score, lactate, leucocyte, lymphocyte, d-dimer and creatinine at the ICU admission, maximum respiratory support, the usage of steroid, IL-6 blocker, and cytokine filter).

The total mortality rate was 27.4%. Mortality rates of GI and GII were 5% and 35.9%, respectively. In GII, major thromboembolic events were not detected in any patients. The median (min-max) day for the starting TPE was 3 (2–4). In GIIa, APACHE II, SOFA scores, d-dimer and interleukin-6 (IL-6) levels at the ICU admission, and length of ICU stay were significantly higher than those of GI whereas mortality rates were similar in those groups (Table 1). The median values of the LOS-ICU in survivors and non-survivors in GII were 14 (6.5–21.5) and 15.5 (8–23), respectively (*p* = 0.630). In GIIa, lactate dehydrogenase (LDH), d-dimer, ferritin, IL-6, C-reactive protein (CRP), and procalcitonin levels were significantly decreased after three consecutive TPEs (Table 2). Furthermore, although ferritin level at the ICU admission was higher in GIIa, the mortality rate in both before and after PSM was higher in GIIb (45.7% and 58.3%) than in GIIa (16.7% and 8.3%) (*p* = 0.037, *p* = 0.009, respectively) (Table 1).

**Table 1:** Comparisons of patients groups

	GI (d-dimer < 2) (*n* = 20)	GII (d-dimer ≥ 2)						
		Before PSM				After PSM		
		GIIa (TPE+) (*n* = 18)	GIIb (TPE−) (*n* = 35)	p_1_ (GI and GIIa)	p_2_ (GIIa and GIIb)	GIIa (TPE+) (*n* = 12)	GIIb (TPE−) (*n* = 12)	p_2_ (GIIa and GIIb)
Age (years)	60 ± 14	62 ± 12	62 ± 15	0.615	0.951	61 ± 14	64 ± 17	0.605
Male, *n* (%)	13 (65.0)	14 (77.8)	26 (74.3)	0.386	0.780	8 (66.7)	8 (66.7)	1.000
BMI (kg/m^2^)	27.3 (5.8)	27.9 (5.5)	27.3 (6.6)	0.290	0.237	28.5 (6.1)	25.0 (6.6)	0.078
CCI	2.5 (4)	3 (3)	4 (3)	0.919	0.422	3.0 ± 2.2	3.8 ± 1.7	0.270
At the ICU admission								
APACHE II	12 ± 4	17 ± 4	17 ± 5	***0.002***	0.886	17 ± 3.3	17.5 ± 5.6	0.794
SOFA Score	5 (3)	6 (1)	7 (3)	***0.002***	0.223	6 (2)	6 (2)	0.713
PaO_2_/FiO_2_ ratio	128 (68)	97 (51)	113 (79)	0.251	0.229	108 (106)	125 (103)	0.551
SpO_2_ (%)	89 (5)	91 (7)	89 (5)	0.377	0.597	92 (10)	91 (5)	0.590
Lactate (mmol/L)	1.4 (0.6)	1.4 (0.7)	1.4 (0.9)	0.988	0.631	1.5 (0.8)	1.3 (0.5)	0.291
WBC (×10^3^/μL)	9.6 (3.9)	6.9 (6.4)	8.2 (6.5)	0.573	0.353	8.7 ± 4.9	7.4 ± 2.7	0.430
Lymc (×10^3^/μL)	0.82 ± 0.40	0.80 ± 0.34	0.89 ± 0.42	0.553	0.271	0.83 ± 0.3	0.82 ± 0.5	0.963
d-dimer (mg/L)^&^	1.2 (0.3–1.9)	5.0 (2.1–35.2)	7.2 (2.1–35.5)	***< 0.001***	0.151	4.5 (2.1–35.2)	6.0 (2.2–32.2)	0.514
Ferritin (ng/mL)	1015 (1735)	1735 (1853)	900 (1454)	0.158	***0.018***	1742 (2117)	605 (1346)	***0.012***
IL-6 (pg/mL)^&^	28.3 (5.3–1418)^(8)^	134 (36.2–2958)^(13)^	254 (33–5233)^(13)^	***0.036***	0.101	155 (39.6–2958)^(8)^	237 (33–4885)^(4)^	0.933
CRP (mg/dL)	18.6 ± 10.9	22.2 ± 12.1	27.8 ± 10.4	0.340	0.086	19.2 ± 10.3	24.0 ± 11.0	0.275
Creatinine (mg/dL)	0.88 (0.29)	0.87 (0.37)	0.99 (0.82)	0.874	0.051	0.91 ± 0.3	0.90 ± 0.3	0.944
Urea (mg/dL)	28 (29)	32 (19)	36 (26)	0.942	0.288	28 (32)	35 (14)	0.291
Number of damaged lobes, *n* (%)^&^	3 (2–4)	3 (2–5)	3 (2–5)	0.149	0.118	3 (2–5)	3 (3–5)	0.671
In the first 48 h								
Breath rate/min (max)	34 (6)	33 (9)	33 (5)	0.988	0.713	33 (11)	33 (5)	0.590
PaO_2_/FiO_2_ ratio (min)	117 ± 42	98 ± 30	105 ± 34	0.087	0.376	104 ± 32.4	120 ± 32.5	0.235
FiO_2_ (%) (max)	75 (48)	80 (30)	80 (35)	0.082	0.969	80 (25)	80 (30)	0.799
PEEP (cmH_2_O) (max)	12 (6)	12 (4)	14 (4)	0.502	0.056	12.0 ± 2.3	13.0 ± 1.9	0.215
C_dyn_ (ml/cmH_2_O) (min)	44 (6)	37 (12)	41 (8)	***0.003***	0.058	36.3 ± 6.6	39.5 ± 7.0	0.265
In the first week								
WBC (×10^3^/μL) (max)	13.2 (5.8)	11.0 (8.9)	12.6 (6.6)	0.077	0.086	10.4 (10.3)	11.0 (6.7)	0.590
WBC (×10^3^/μL) (min)	5.9 (2)	6.3 (4)	4.9 (4)	0.718	0.612	6.7 (4.4)	4.6 (1.5)	0.219
Lymc (×10^3^/μL) (min)	0.48 (0.40)	0.5 (0.28)	0.49 (0.46)	0.919	0.573	0.52 (0.29)	0.45 (0.28)	0.551
NLCR (max)	16.4 (16.2)	15 (8)	11 (11)	0.460	0.517	13.6 (10.1)	11.6 (11.5)	0.843
Lactate (mmol/L) (max)	2.1 (0.7)	2.4 (1.1)	2.4 (0.8)	0.087	0.955	2.3 (1.0)	2.4 (1.6)	0.347
Fluid balance (mL)	3670 (3198)	4552 (2973)	3849 (2196)	0.874	0.441	4174 ± 2907	5331 ± 3170	0.361
Total fluid (mL/kg/day)	40.7 (9.3)	44.3 (15.5)	44.8 (11)	0.696	0.910	44.8 ± 13.5	48.7 ± 12.0	0.460
Respiratory support (max), *n* (%)								
IMV	13 (65.0)	16 (88.8)	30 (85.7)	0.084	0.746	11 (91.7)	12 (100)	0.307
NIMV	3 (15.0)	1 (5.6)	3 (8.6)	0.344	0.694	1 (8.3)	0	0.307
HFOT	4 (20.0)	1 (5.6)	2 (5.7)	0.188	0.981	0	0	NA
Additional therapies, *n* (%)								
Cytokine filters	1 (5.0)	3 (16.7)	3 (8.1)	0.427	0.434	2 (16.7)	1 (8.3)	0.592
IL-6 blocker	12 (60.0)	9 (50.0)	20 (57.1)	0.536	0.621	7 (58.3)	6 (50)	0.682
Steroids	11 (55.0)	10 (55.6)	20 (57.1)	0.357	0.912	7 (58.3)	7 (58.3)	1.000
Duration of IMV (h)^&^	168 (0–816)	286 (0–1008)	192 (0–720)	0.1 12	0.067	316 ± 271	278 ± 139	0.671
AKI, *n* (%)	7 (35.0)	6 (33.3)	19 (54.3)	0.914	0.148	3 (25)	6 (50)	0.206
Tracheotomized patients, *n* (%)	2 (10.0)	2 (11.1)	1 (2.9)	0.911	0.218	1 (8.3)	0 (0)	0.307
LOS-ICU, (days)^&^	12 (6–34)	20 (5–42)	11 (7–35)	***0.017***	***0.003***	20 ± 10	14 ± 5	0.067
Mortality, *n* (%)	1 (5.0)	3 (16.7)	16 (45.7)	0.242	***0.037***	1 (8.3)	7 (58.3)	***0.009***

*AKI* acute kidney injury, *APACHE II* Acute Physiology and Chronic Health Evaluation, *BMI* body mass index, *CCI* Charlson comorbidity index, *C*_*dyn*_ dynamic compliance, *CRP* C-reactive protein, *HFOT* high-flow oxygen therapy, I*CU* intensive care unit, *IL-6* interleukin-6, *IMV* invasive mechanical ventilation, *LOS* length of stay, *Lymc* lymphocyte count, *NIMV* non-invasive mechanical ventilation, *NLCR* neutrophil-lymphocyte count ratio, *PSM* propensity score matching, *SOFA*, sequential organ failure assessment, *TPE* therapeutic plasma exchange, *WBC* white blood cell. Results were given as percentage, mean ± sd, and median (IQR or min-max). ^&^Minimum and maximum values. Student *t* and Mann-Whitney *U* tests were used for statistical analysis

**Table 2:** Comparisons of laboratory parameters in pre and post-TPE procedure

	Pre-TPE	Post-TPE	*p*
WBC (× 10^3^/μL)	9.08 ± 4.1	9.14 ± 3.5	0.951
Neuc (×10^3^/μL)	7.38 ± 3.1	7.33 ± 3.3	0.953
Lymc (× 10^3^/μL)	0.9 (0.5–1.3)	1.02 (0.77–1.27)	0.053
NLCR	6.8 (1.8–11.7)	6.7 (4.2–9.2)	0.184
LDH (IU/L)	436 (322–550)	239 (181–297)	***0.001***
d-dimer (mg/L)^&^	7.8 (2.1–35.2)	1.3 (0.6–3.9)	***< 0.001***
Ferritin (ng/mL)^&^	1268 (399–6110)	405 (157–1650)	***0.001***
IL-6 (pq/mL)^(13)&^	161 (36.2–2958)	24.5 (1.5–130)	***0.001***
CRP (mg/dL)^&^	11.8 (0.4–29.7)	0.9 (0.3–7.2)	***< 0.001***
Procalcitonin (ng/mL)^&^	0.27 (0.02–87)	0.1 (0.01–39)	***0.002***

*CRP* C-reactive protein, *IL-6* interleukin-6, *LDH* lactate dehydrogenase, *Lymc* lymphocyte count, *Neuc* neutrophil count, *NLCR* neutrophil-lymphocyte count ratio, *TPE* therapeutic plasma exchange, *WBC* white blood cell. Results were given as percentage, mean ± sd, and median (quartiles or min-max). ^&^Minimum and maximum values. Paired sample and Wilcoxon tests were used for the statistical analysis

Some patients with COVID-19 pneumonia have a high risk of thrombosis leading to worse outcomes. Therefore, monitoring d-dimer levels is crucial. In these groups of patients, TPE seems to be a treatment which may improve outcomes by effectively removing FDPs and restoring coagulation status. We are aware that TPE may not be routinely required in these patients [6]. However, we think that it should be featured as a part of the treatment especially in COVID-19 pneumonia patients with a high risk of thrombosis.

## Data Availability

All data was added as an Excel file in a supplementary information file.
